# Regeneration of the digestive tract of an anterior-eviscerating sea cucumber, *Eupentacta quinquesemita*, and the involvement of mesenchymal–epithelial transition in digestive tube formation

**DOI:** 10.1186/s40851-019-0133-3

**Published:** 2019-06-21

**Authors:** Akari Okada, Mariko Kondo

**Affiliations:** 1 Misaki Marine Biological Station, Graduate School of Science, The University of Tokyo, 1024 Koajiro Misaki, Miura, Kanagawa 238-0225 Japan; 2 Center for Marine Biology, The University of Tokyo, 1024 Koajiro Misaki, Miura, Kanagawa 238-0225 Japan; 3 Laboratory of Aquatic Molecular Biology and Biotechnology, Graduate School of Agricultural and Life Sciences, the University of Tokyo, 1-1-1 Yayoi, Tokyo, 113-8657 Bunkyo Japan

**Keywords:** Echinoderm, Sea cucumber, Regeneration, Digestive tract, Evisceration, Mesentery, Mesenchymal–epithelial transition

## Abstract

Sea cucumbers (a class of echinoderms) exhibit a high capacity for regeneration, such that, following ejection of inner organs in a process called evisceration, the lost organs regenerate. There are two ways by which evisceration occurs in sea cucmber species: from the mouth (anterior) or the anus (posterior). Intriguingly, regenerating tissues are formed at both the anterior and posterior regions and extend toward the opposite ends, and merge to form a complete digestive tract. From the posterior side, the digestive tube regenerates extending a continuous tube from the cloaca, which remains at evisceration. In posteriorly-eviscerating species, the esophagus remains in the body, and a new tube regenerates continuously from it. However, in anterior-eviscerating species, no tubular tissue remains in the anterior region, raising the question of how the new digestive tube forms in the anterior regenerate. We addressed this question by detailed histological observations of the regenerating anterior digestive tract in a small sea cucumber, *Eupentacta quinquesemita* (“ishiko” in Japanese) after induced-evisceration. We found that an initial rudiment consisting of mesenchymal cells is formed along the edge of the anterior mesentery from the anterior end, and then, among the mesenchymal cells, multiple clusters of epithelial-like cells appears simultaneously and repeatedly in the extending region by mesenchymal–epithelial transition (MET) as visulalized using toluidine blue staining. Subsequently, multiple cavities were formed surrounded with these epithelial cells, and appeared to coalesce with each other to form into multiple lumens, and to eventually become a single tube. This anterior tube then fused to the tube regenerated from the posterior rudiment. Thus, we elucidated the process of regeneration of the anterior portion of the gut in an anteriorly eviscerating species, and suggest the involvement of MET and fusion of cavities/lumens in regeneration of the digestive tube.

**Electronic supplementary material:**

The online version of this article (10.1186/s40851-019-0133-3) contains supplementary material, which is available to authorized users.

## Background

Regeneration is the re-growth of missing or damaged tissues or organs in adults or even in larvae or embryos, and often comparable to embryogenesis in that they involve morphogenesis. In embryogenesis, the whole embryo develops various tissues in a coordinated manner, whereas during regeneration, an animal that lost a part of their body restores that part only without affecting other parts. Thus, regeneration is a self-organizing phenomenon, and its underlying mechanism may differ from that of normal embryogenesis. Although many studies have been published, the field of regeneration research has many unanswered questions, such as how regeneration is possible, what molecules are triggers for regeneration, and why the ability to regenerate differs among animals [1, 2].

Various animals are known for their high ability to regenerate and are used for regeneration studies, for example, planarians and newts. Although they receive less attention in this context, echinoderms, consisting of five classes, regenerate efficiently and are also the subject of study [3–8]. For example, sea stars, brittle stars, and crinoids have high potential for regeneration, and are able to self-amputate (autotomize) their arms, but then are able to completely regrow them. Some sea stars are known to regenerate an entire body even from an arm, and some crinoids also regenerate their viscera (reviewed in [8]).

Sea cucumbers are known to exhibit extensive regenerative ability and regenerate a wide spectrum of body parts, e.g. their body wall, the nervous system, and internal organs, such as the digestive system, reproductive organs, and respiratory trees. Ejection and regeneration of the sea cucumber digestive system has been the object of studies, and observations have been documented for more than 100 years ([9] and references therein). Evisceration, the ejection of almost the entire set of internal organs, happens under natural conditions ([9] and references therein), as well as in response to unnatural or experimental environmental cues, such as foul water or external mechanical or chemical stimuli. In evisceration, the digestive tract first autotomizes at the posterior or anterior ends, and is ejected from either the mouth (anterior evisceration) or the anus (posterior evisceration). Sea cucumbers of the orders Dendrochirotida (sea cucumbers that have branched tentacles and are mostly filter-feeders) and Aspidochirotida (sea cucumbers with flattened tentacles) are known to eviscerate. Dendrochirotid and aspidochirotid sea cucumbers mainly eviscerate anteriorly or posteriorly, respectively [10, 11]. In either case, the mesentery remains, which connects the internal lining (mesothelium) of the body cavity and the digestive tract. Animals that eviscerate anteriorly release the entire digestive tract, except for the cloaca, and loses the entire set of anterior end structures, such as the oral (aquapharyngeal) complex and the tentacles. Those that eviscerate posteriorly release the intestine between the esophagus and the cloaca. During regeneration, despite the differences in type of evisceration, the free edge of the mesentery thickens, which contributes to the gut primordium. In posterior eviscerating sea cucumbers, the wound at the end of the remaining esophagus and cloaca due to autotomy are closed and anterior and posterior blind gut rudiments are formed, and these tubes grow and eventually join to form a continuous digestive tract [12, 13]. By contrast, in anterior eviscerating species, a blind tube is formed continuously from the remaining cloaca, but in the anterior part, a rudiment of a mass of cells is first formed and then the tube somehow regenerates and extends posteriorly. This phenomenon has been studied in the dendrochirotid sea cucumber, *Eupentacta fraudatrix*, as an example [14]. Based on histological data, it has been reported that a rudiment consisting of a solid rod of connective tissue covered by coelomic epithelium appears at the oral end of the body. According to Mashanov et al. (2005) [14], “dedifferentiated mesothelium” covering the ventral side of the rudiment developed “folds that penetrate the underlying connective tissue,” and after several days of regeneration, this “epithelial lining of the folds detaches from the surface” and forms “a number of lumina that fuse into a single blind lumen lined with a newly formed luminal epithelium,” which is “derived from the external mesothelium of the rudiment,” and they propose transdifferentiation of coelomic epithelium into digestive epithelium. If this is the case, the mesothelium of mesodermal origin transdifferentiates into the digestive epithelium of endodermal origin, while retaining epithelial structure. No other study reports the direct trasndifferentiation of mesodermal epithelium into endodermal epithelium, as far as we know, although there are studies in sea cucumber [15] and feather star [16] that suggest endodermal structures could originate from mesodermally derived cells. This raised questions about the results reported in Mashanov et al. (2005), since it was not clearly shown in their data in what manner invagination occurs, now how or if multiple lumens are formed, and how they fuse to form a single lumen. We thus addressed this question in this study using a related sea cucumber, *Eupentacta quinquesemita*, known as “ishiko” in Japanese*.*

**Fig. 1:**
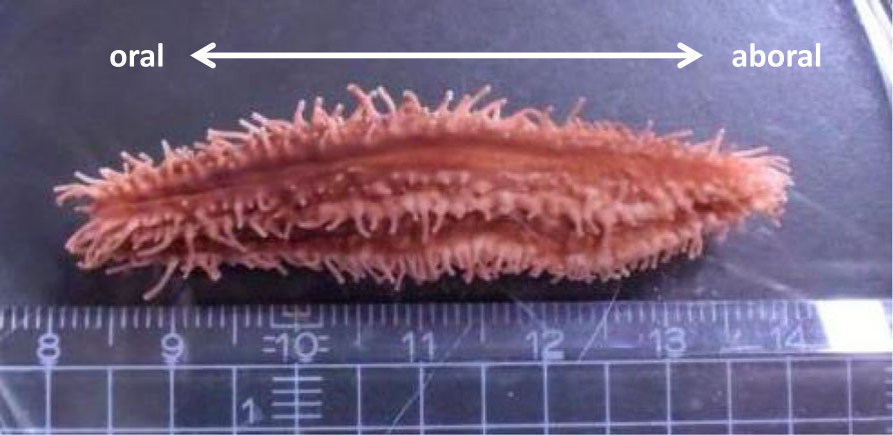
The animal used in the study. A small sea cucumber, *Eupentacta quinquesemita*, is about 5 cm in size. The left side in this figure is oral (anterior), and the right side is aboral (posterior)

*Eupentacta quinquesemita* (ishiko) (Fig. 1) is a small sea cucumber that belongs to same genus as the species handled in previous studies on intestine regeneration [13, 14]. Morphological observation of regenerating intestines of wild-caught ishiko has been reported [17]. This species eviscerates anteriorly and seasonally in nature [18, 19]. Evisceration may be induced experimentally by chemicals such as KCl [20], and the presence of an endogenous “evisceration factor” has been reported [21]. Using induced evisceration, the morphology of the sites of autotomy has been studied [22].

The digestive tract of ishiko is made up of the oral complex, stomach, intestine and cloaca, and they are anchored to body wall by the mesentery (Additional file 1: Figure S1a). The intestines can be divided into three parts: the first descending intestine, the ascending intestine, and the second descending intestine. When evisceration occurs, autotomy happens at four regions between 1) the oral body wall/the oral complex (the introvert), 2) the longitudinal muscle/the retractor muscle, 3) intestine/mesentery and 4) intestine/cloaca, and, after evisceration, the mesentery and cloaca remain in the body cavity [14, 22]. The general structure of the mesentery of sea cucumbers has been described by Hyman (1955) [23] as subdivided into three parts. We have dissected ishiko, and similarly, the three parts of the mesentery was observed. The mesentery does not run straight along one side of the body wall, but is connected to the dorsal side near the mouth and ends on the ventral side. The first part is an anterior part that attaches to the dorsal body wall, connected to the oral complex up to the middle part of the intestine near the gonads (1st descending intestine) (Additional file 1: Figure S1b). The second middle part curves to the left and anterior side, supporting the ascending intestine, up to the ventral side of the body wall. The direction of the mesentery then changes to the posterior, and this third part of the mesentery supports the 2nd descending intestine and the cloaca, attached to the ventral side of the body (Additional file 1: Figure S1c). Due to the curvature of the mesentery, when the animal is dissected open and flattened, the mesentery appears to be in a S-shape (Additional file 1: Figure S1a).

In a trial to induce evisceration in ishiko, we confirmed that oral tissues were lost. These results were consistent with previous studies (for example, [22]). As mentioned earlier, evisceration in this species has already been reported, but to our knowledge, there are no descriptions on the process of regeneration, except for the external morphology of the regenerating digestive tract following seasonal evisceration [17]. In order to provide insight into the formation of the regenerating anterior digestive tract in ishiko, we performed histological observations during the course of regeneration.

## Materials and methods

### Animals

The sea cucumber, *Eupentacta quinquesemita* was collected by diving at the depth of about 3 m in Sagami Bay, near the pier of Misaki Marine Biological Station in Miura, Kanagawa Prefecture, or at the depth of about 5–10 m near Aquamarine Fukushima in Onahama, Fukushima Prefecture. Animals of body lengths between 5 and 10 cm were kept in tanks until used for experiments. Referring to Byrne (1986) [21], evisceration was induced by injection of approximately 100 μL of 0.45 M KCl into the coelom. Eviscerated animals were returned into aquaria with sea water at 13–18 °C without feeding, up to 3 weeks, to allow them to regenerate. Animals were collected every few days for examination. The day of evisceration was designated as 0 days post evisceration (dpe). For each series of experiments, multiple individuals were induced to eviscerate simultaneously, and several animals were used for observation at various stages of regeneration.

### Morphological and histological observations

Animals were anesthetized in 72 g/L MgCl_2_ in sea water for approximately 15 min to 1 h before dissection. They were dissected along the body length at the right ventral interambulacral zone, exposing the whole body cavity, and examined under a stereomicroscope. In total, 86 eviscerated animals were used for morphological observations, of which 20 were used for histological observations. Additionally, several intact adult animals were studied. After categorizing the progress of regeneration into four stages, we investigated histologically the regenerating tissues of one specimen at stage I, three at stageII, 13 at stageIII, and three at stage IV. Gonads that lie over the intestine and mesenteries were removed. The mesentery with regenerating tissues were cut out with the piece of the body wall where it was attached, and fixed in Bouin’s Fixative overnight at room temperature. Subsequently, the fixative was replaced with 70% ethanol and the specimens were stored at room temperature until embedding in paraffin (Paraplast X-TRA, SIGMA) for histological observation. Serial sections of 6–10 μm thickness were produced, mostly perpendicular to the anterior-posterior (oral-aboral) axis of the body. They were stained with hematoxylin-eosin (HE) or toluidine blue (TB) and observed on a light microscope (ECLIPSE TE300, Nikon). TB was chosen since it is well known that this stain binds acidic components such as acidic mucopolysaccharides and show metachromasia. Through the course of our experiments, we found that staining with TB is better than HE for distinguishing cell types, namely between epithelium-like cells and mesenchyme.

## Results

### Morphological observation of the regenerating digestive tract

When *Eupentacta quinquesemita* (ishiko) was induced to eviscerate by the injection of KCl, the internal organs were ejected from the mouth within about 15 min. The oral complex, the digestive tract, and part of the gonads were ejected anteriorly (Additional file 2: Figure S2), leaving a wound opening at the anterior tip.

**Fig. 2:**
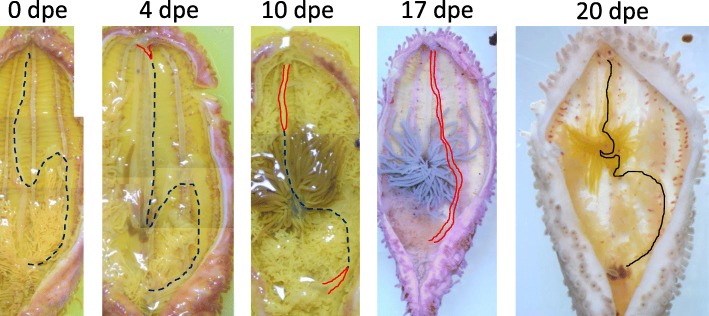
Internal morphology of regenerating animals. Animals just after evisceration (0 dpe) to 20 dpe were dissected along the body length, at the right side of the mid-ventral ambulacrum and flattened. There are slight color variations of the animals, but specimens at 0, 4, and 10 dpe were fixed with Bouin’s fixative and thus appear yellowish. The animals were about 4–5 cm in length. Black broken line: the edge of mesentery; red solid line: area along the edge of the mesentery, with thickened gut rudiments; black line: grown gut rudiment observable without the microscope

**Fig. 3:**
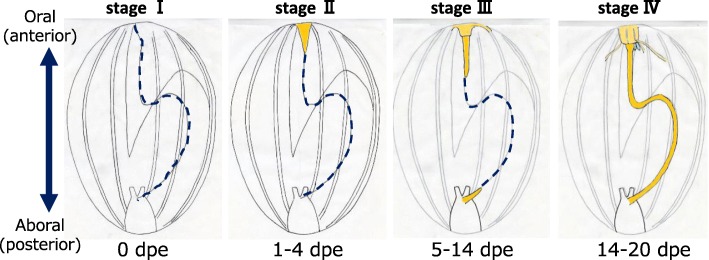
Stages of regeneration. Based on the internal morphology of regenerating animals, we defined four stages of regeneration, stages I to IV, and schematic diagrams of each are shown. Blue broken line: the edge of mesentery without regenerating tissues; yellow colored area: regenerating tissues (gut rudiment)

To record the progress of regeneration, the animals were dissected and the internal morphology was observed (Fig. 2). The degree of regeneration of the digestive tract was not the same in all animals, i.e., some individuals regenerated faster than the others, so based on morphology, we divided the process into 4 stages, stages I to IV (Fig. 3). It took 2–3 weeks until a continuous gut rudiment was formed after evisceration.

#### Stage I (just after evisceration)

Soon after evisceration, we observed that the hole (wound) made in the anterior end of the body was closed by contraction of the body wall. In the body cavity, only the mesenteries and cloaca remained (Fig. 2, 0dpe). The edge of the mesenteries that had been in connection with the digestive tract was free. Some gonadal tissues, the gonadal duct (embedded in the mesentery), and respiratory trees were also left in the body cavity. These features were observed 0–1 dpe.

#### Stage II (formation of anterior regenerating tissues)

In a specimen at 4 dpe (Fig. 2, 4 dpe), the oral wound was closed. The anterior tips of the pentaradial longitudinal muscles and radial water canals were converged at the anterior end of the body, and formed a mass of cells that appeared as a thickening (depicted in yellow in Fig. 3, Stage II). The wound was completely closed. This thickening is potentially the rudiment from which the oral complex and the digestive tract regenerate, and is termed the “anterior rudiment” in Mashanov et al. (2005) [14]. In contrast, no cell mass nor thickening of the mesentery were observed at the region where the cloaca was attached to the mesentery. Similar features were observed in animals 1–4 dpe.

#### Stage III (formation of posterior regenerating tissues and growth of the anterior regenerating tissues)

We observed in a specimen at 10 dpe that regenerating tissues in a rod-like shape extended from the anterior rudiment (Fig. 2, 10 dpe). This tissue was connected to and appeared to elongate along the free edge of the mesentery. On the posterior side, a thickening of the edge of the mesentery was observed. At the free edge of the middle part of the mesentery, no evident thickening was observed, and the anterior and posterior gut rudiments (depicted in yellow in Fig. 3, Stage III) were separated. Similar features were observed in other specimens between 5 and 14 dpe.

#### Stage IV (formation of a continuous rudiment)

The presence of a continuous rudiment was observed between 14 and 20 dpe, depending on the specimen. In one specimen at 17 dpe (Fig. 2, 17 dpe), a thickening of the edge was observed in the entire mesentery, indicating that the anterior and posterior regenerating tissues are connected. The continuous regenerating tract appeared straight without curving, although the mesentery is attached to the body wall in a S-shaped curve. It has been reported in another sea cucumber species, *Thyone okeni*, that the mesentery grows wider during the course of regeneration [24], and this allows the rudiment to take a short route between the mouth and the anus. In another specimen at 20 dpe (Fig. 2, 20 dpe), the gut rudiment had thickened and elongated to be observable without a microscope, although it was still smaller than an intact one. The middle part of the rudiment expected to become the 1st descending intestine to the ascending intestine was looped. After this stage, the connected rudiment of the digestive tract grew further, forming loops.

### Histological observation of the intact digestive tract

**Fig. 4:**
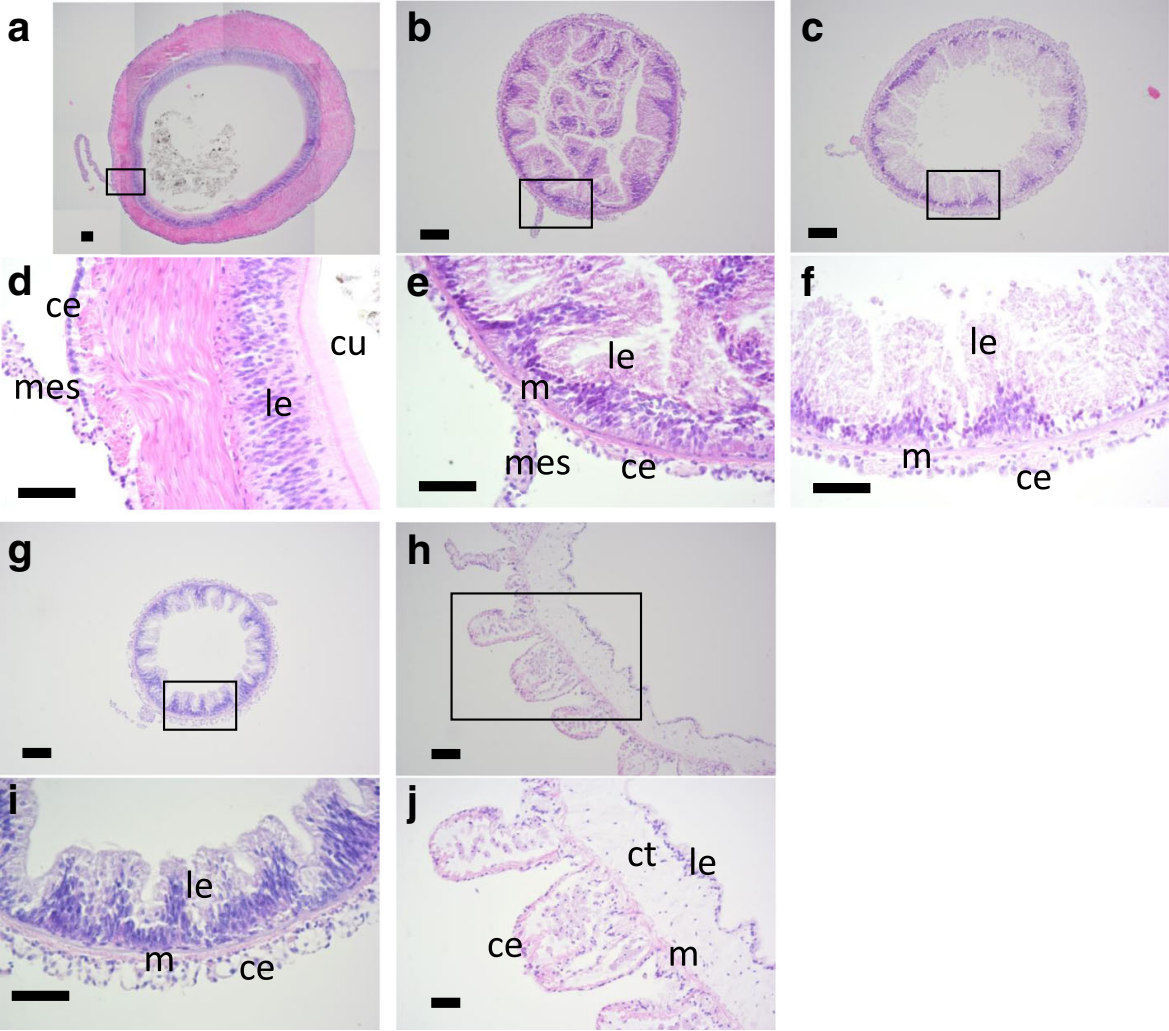
Histology of the intact digestive tract. Cross sections of the digestive tract of *E. quinquesemita* were stained with HE. **d**, **e**, **f**, **i**, **j** are high-magnification views of the boxed areas in **a**, **b**, **c**, **g**, **h**, respectively. **a**, **d** Stomach. Some contents of the stomach are also observed in **a**. **b**, **e** 1st descending intestine. **c**, **f** Ascending intestine. **g**, **i** 2nd descending intestine. **h**, **j** Cloaca. ce, celomic epithelium; ct, connective tissue; cu, cuticle; gd, gonadal duct; le, luminal epithelium; m, muscle; mes, mesentery. Scale bars100 μm in **a**-**c**, **g**, **h**; 50 μm in **d**-**f**, **i**, **j**

The intact digestive tract is mainly composed of four tissue types: the coelomic epithelium, muscle, connective tissues and the luminal epithelium. The tissue structure differed depending on the part of the digestive tract. In the stomach the muscle layer developed well and luminal epithelium was covered with cuticles (Fig. 4a,d). The muscle layers of the three parts of the intestine were thinner than the stomach and the luminal epithelia was folded (Fig. 4b, c, e, f, g, i). The luminal epithelia of the 1st descending intestine was thicker than the other two parts of the intestine. We observed additional luminal epithelia in the lumen of the thin sections, which we interpret as the result of the luminal epithelium extending into the lumen and folding (Fig. 4b). The cloaca is linked to body wall with tendons, and at the sites of the connection, there were protrusions on the coelomic side, which consisted of coelomic epithelium and connective tissues. A thin layer of muscles, thick connective tissues and smooth luminal epithelium were observed as layers (Fig. 4h,j).

### Histological observation of the regenerating tissues

We investigated histologically the regenerating intestinal tissues at each stage of regeneration.

#### Stage I

**Fig. 5:**
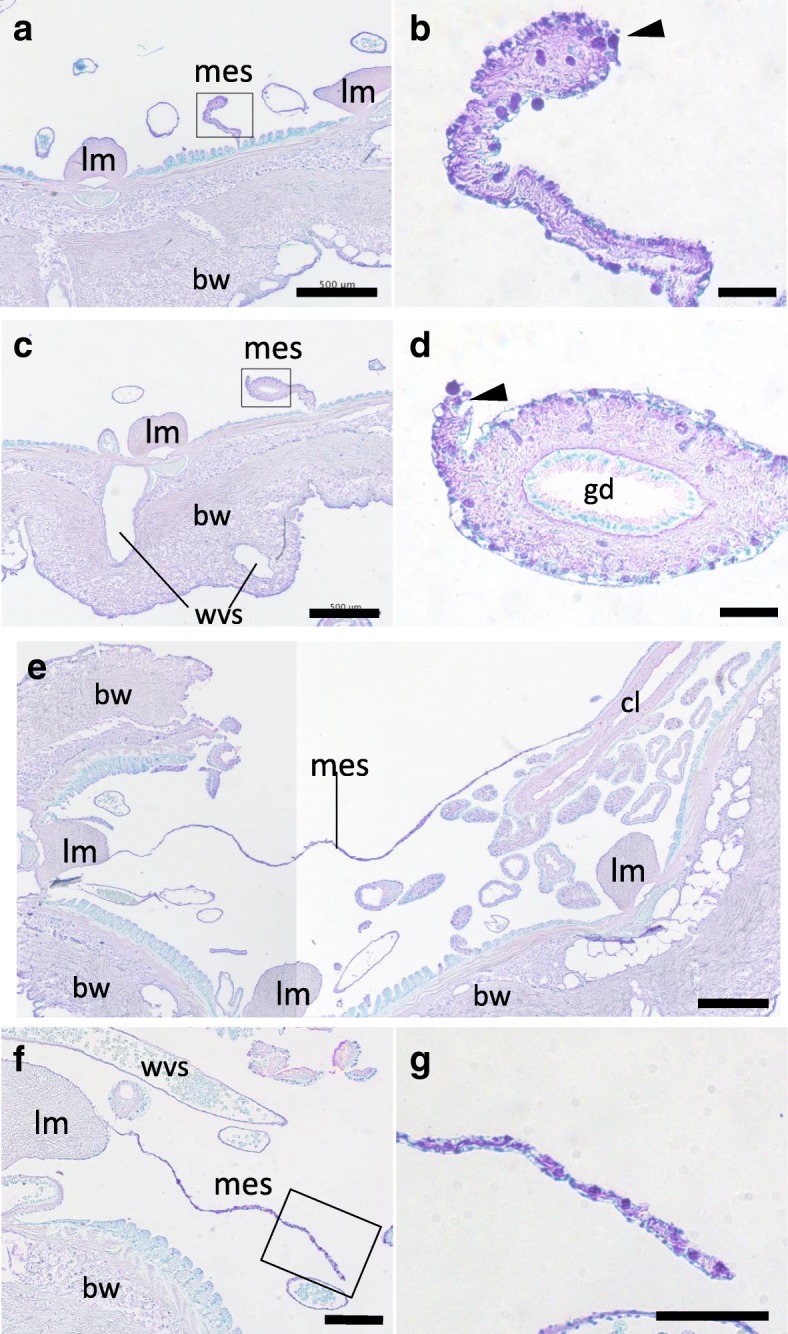
Histology of the tissues at stage I. Cross sections of the body at stage I (0 dpe) were stained with TB, mainly focusing on the anterior (**a**-**d**) and posterior (**e**, **f**) mesenteries. **b**, **d** and **g** are high-magnification views of the boxed areas in **a**, **c** and **f**, respectively. **a**, **b** Anterior tissue, posterior to the anterior rudiment. **c**, **d** Anterior tissue posterior than **a** and **b**. The gonadal duct remains in the mesentery, but no structures are present at the free end. **e** Posterior tissue. The cloaca and the body wall are connected by the mesentery. **f**, **g** Posterior tissue, anterior than **e**. In **b**, **d** and **g**, no tubular structures nor developed cell mass are observed at the free edge of the mesentery (arrowhead). bw, body wall; cl, cloaca; gd, gonadal duct; lm, longitudinal muscle; mes, mesentery; wvs: a part of the water vascular system (radial water canals, etc.). Arrowheads indicate the free edges of the mesentery. Scale bars 500 μm in **a**, **c**, **e**; 200 μm in **f**; 50 μm in **b**, **d**, **g**

In the specimen about 1 to 1.5 h after evisceration, only the mesentery and cloaca remained in the body cavity. The mesentery at the oral side was thin, though the free edge appeared to be wider than closer to the body wall (Fig. 5a, b), and no cell mass or thickening that was observed in later stages were present. The gonadal duct is embedded in the mesentery, between its distal end where the intact digestive tract is attached and the body wall (Additional file 1: Figure S1b). The gonadal duct survives evisceration except for the segment that had been connected to the oral complex, and this was confirmed (Fig. 5c, d). The intestine detached from the posterior mesentery, anterior to the cloaca, and did not show any mass of cells at the free edge (Fig. 5e, f, g).

#### Stage II

**Fig. 6:**
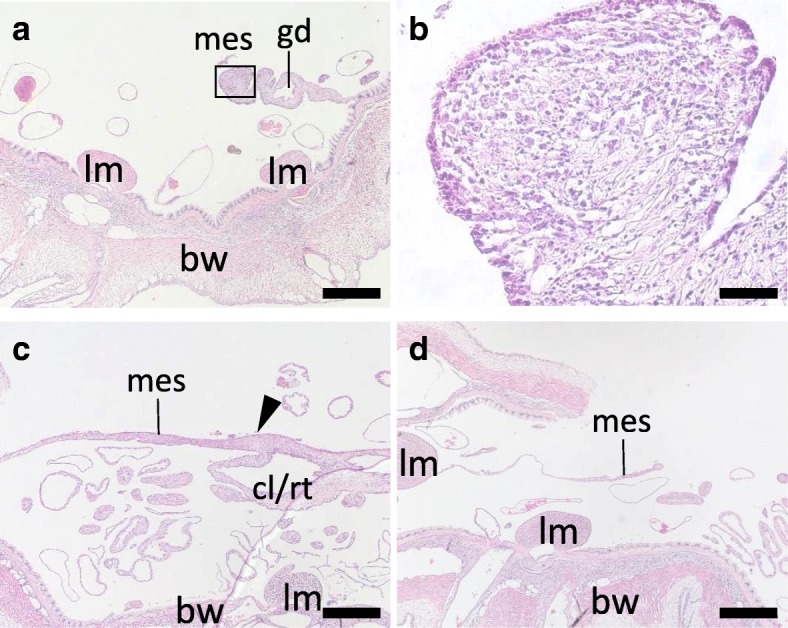
Histology of the tissues at stage II. Cross sections of the body at stage II (4 dpe) were stained with HE, focusing on the mesentery at the anterior (**a**, **b**) and posterior (**c**, **d**) regions of the body. **a**, **b** Anterior mesentery. A mass of cells is present on the free edge of the mesentery. **b** is a high-magnification view of the boxed area in **a**. **c** Posterior tissue. A thickening of tissue (arrowhead) is observed at the connection of the mesentery and the cloaca. Respiratory trees are an organ continuous from the cloaca, and thus these two tissues could not be strictly distinguished. **d** Posterior tissue, anterior than **c**. A part of the mesentery with a free edge is shown. bw, body wall; cl/rt., cloaca or respiratory trees; gd, gonadal duct; lm, longitudinal muscle; mes, mesentery. Scale bars 500 μm in **a**, **c**, **d**; 50 μm in **b**

As described earlier, according to morphological observation at this stage, thickening of tissues on the free edge of the mesentery was observed on the anterior side. The thickening of the free edge of the anterior mesentery was confirmed with histological observation to be a mass of cells (Fig. 6a). The coelomic epithelium surrounding the regenerating tissue (gut rudiment) appeared strongly stained with hematoxylin than the inside (Fig. 6b). Inside of the regenerate, no specific structures were observed, and the cells were mesenchymal. At the posterior side, the part of the mesentery where it connected with the cloaca appeared wider (Fig. 6c). We were not able to tell if this was the gut rudiment that regenerates continuously from the cloaca. In a region of the mesentery closer to the anterior side, we did not observe any regenerating tissue at the edge (Fig. 6d).

#### StageIII

**Fig. 7:**
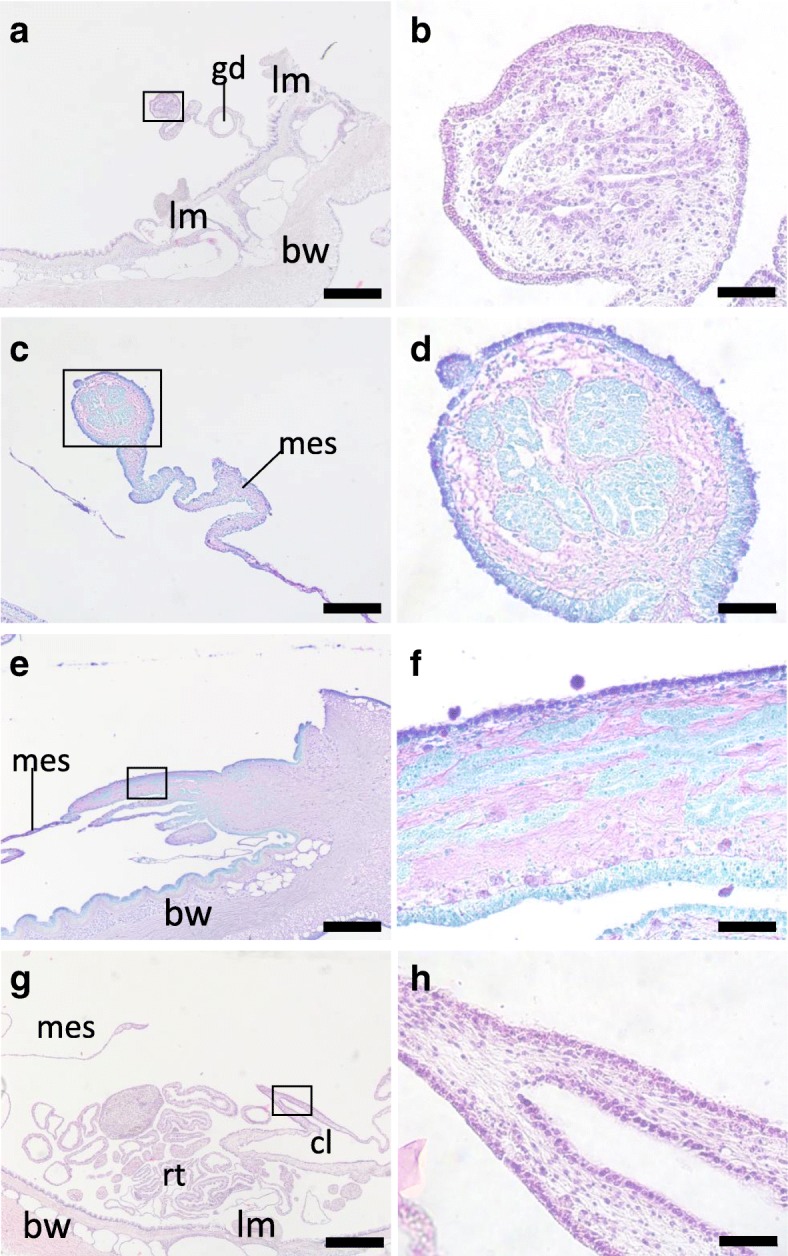
Histology of the tissues at stage III. Histological sections of the anterior (**a**-**f**, **i**) and posterior (**g**, **h**) regions of the body at stage III. **b**, **d**, **f**, **h** are high-magnification views of the boxed areas in **a**, **c**, **e**, **g**, respectively. **a**, **b** Cross section, 6 dpe, stained with HE. **c**, **d** Cross section, 7 dpe, stained with TB. **e**, **f** Longitudinal section of the oral region, 7 dpe, stained with TB. The right side of this figure is anterior. **g**, **h** Posterior region, 6 dpe, stained with HE. The animal was sliced perpendicular to the oral-aboral axis but a longitudinal section of the regenerating tube was made. In the lumen, a mass of obscure material is observed (arrowhead). bw, body wall; cl, cloaca; gd, gonadal duct; mes, mesentery; rt., respiratory trees. Scale bar 500 μm in **a**, **e**, **g**; 200 μm in **c**; 50 μm in **b**, **d**, **h**, **f**

For the observation of this stage, specimens at 6, 7 and 12 dpe were used. To what extent regeneration progressed does not always match the period of regeneration and we did not detect clear differences between the 6 and 7 dpe specimens. In all specimens, thickened tissues were observed at both the anterior and posterior mesenteries in both specimens. The anterior regenerating tissue elongated posteriorly and became thicker in more anterior regions (Fig. 7a). In the regenerated tissues that had grown larger, multiple cavities were found (Fig. 7b). The cells in the anterior regenerate did not show uniform staining intensities with HE, and the cavities were surrounded with cells that were stained stronger than the others (Fig. 7b). TB staining of another specimen revealed that most of the mesenchyme of the regenerate was stained reddish purple and the cells surrounding the lumens were stained light blue (Fig. 7c, d). Cells stained light blue were also found scattered among mesenchymal cells (Fig. 7d). The coelomic epithelium of the regenerate was stained purple and some tissues basal to the epithelium were colored light blue (Fig. 7d). This light blue tissue was observed more on the side closer to the mesentery, and may reflect differences in tissue differentiation. Sagittal sections of another specimen revealed that the light blue-colored tissues surrounding the cavities existed in the regenerating tissue posterior to the regenerating oral complex (Fig. 7e). The cavities were not in contact with the coelomic epithelium and the cavities extended in the anterior-posterior direction independently or possibly fused to each other (Fig. 7f). We observed that on one side of the regenerating tissue the coelomic epithelium was stained in purple whereas the on the other side a layer of cells were stained light blue (Fig. 7f). We are uncertain why a purple layer of cells is not detectable in this figure, but together with Fig. 7d, the data suggest that there may be a difference in differentiation among the cells/tissues in the periphery of the regenerate at the same distance from the anterior end of the body. On the aboral side, a hollow tube was found, extending from the cloaca (Fig. 7g). The luminal epithelium was also continuous from the cloaca. The tissue in the regenerating tissue other than the coelomic and luminal epithelia were mesenchymal (Fig. 7h).

#### Stage IV

**Fig. 8:**
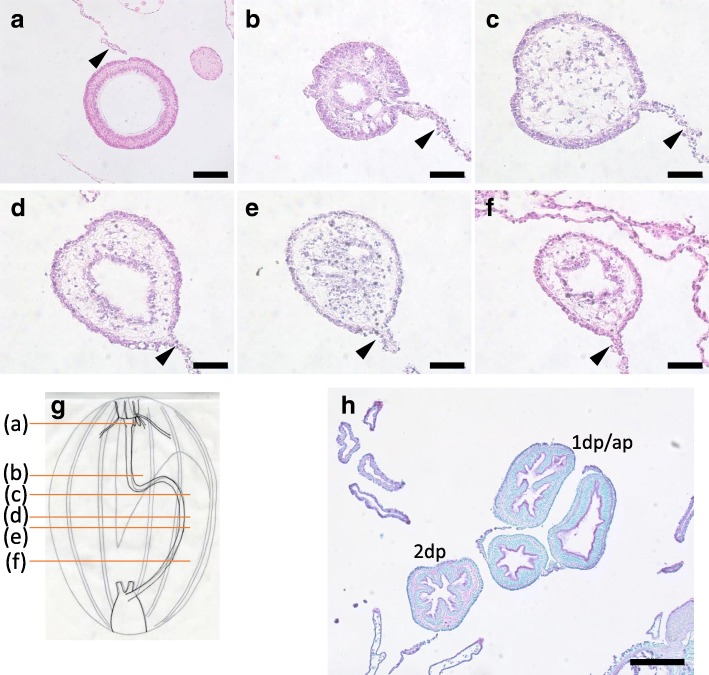
Histology of the tissues at stage IV. Cross sections of the body at stage IV focusing on the regenerated digestive tract. Sections **a**-**f** were made at positions indicated in **g**, of a specimen at 17 dpe. **h** The middle part of the animal with 1st descending, ascending and 2nd descending intestines, at 20 dpe, stained with TB. The slice was made roughly between **b** and **c** in a further regenerated animal with a single tube throughout the digestive tract. The 1st descending and ascending intestines could not be distinguished, since the intestines meandered. 1st descending or ascending intestine; 2dp, 2nd descending intestine. Arrowheads indicate mesenteries. Scale bar 50 μm in **b**-**f**; 100 μm in **a**; 200 μm in **h**

At this stage, the rudiment of the digestive tract was continuous by the elongation and fusion of the both anterior and posterior tissues (Fig. 8g). However, not all regenerating sea cucumbers formed continuous tubes. We observed in a specimen at 17 dpe that in the anterior portion of the regenerate a single lumen was formed (Fig. 8a). There was a layer of cuticle in this tissue that was expected to become the stomach (Figs. 8a and 4a, d). This suggests that differentiation of different regions of the digestive tract occurs as the lumen is formed. Posteriorly, there were regions that had multiple cavities/lumens (Fig. 8b and e), no obvious cavities (Fig. 8c), or a clear single lumen (Fig. 8d), and close to the cloaca in the posterior portion of the regenerate, a single lumen was formed inside (Fig. 8f). In a further regenerated specimen at 20 dpe, a single lumen penetrated the digestive tract from the anterior to posterior end like in intact animal. In the middle of the digestive tract, the intestine meandered and we were observed a histological section of different regions in the same view (Fig. 8h), although we could not distinguish the 1st descending and ascending intestines, since the loops zigzagged. The tissues were not identical to the intact intestine, but some folds in the luminal side were observed, as well as a difference in tissue compositions between the 1st descending and ascending intestines versus the 2nd descending intestine, indicating that the intestines have differentiated or in the process of differentiation.

**Fig. 9:**
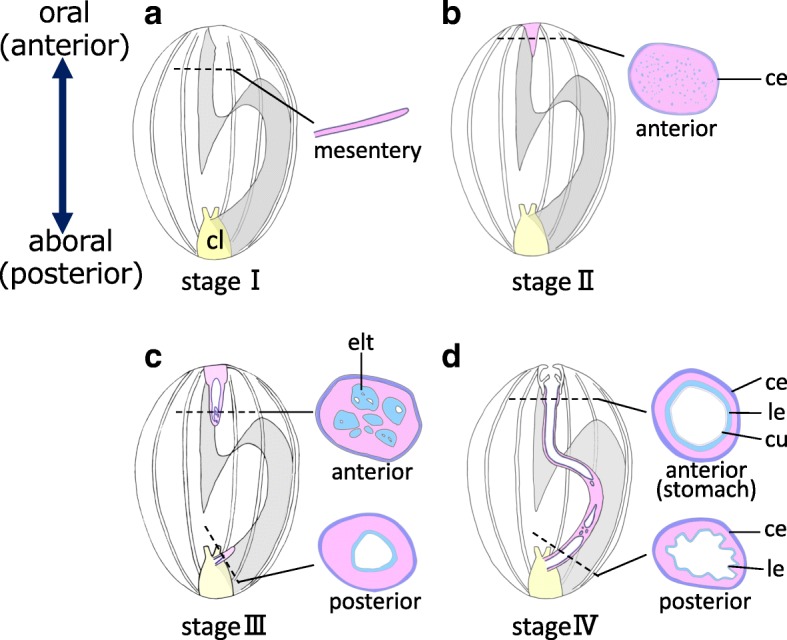
Schematic diagram of regeneration of the digestive tube. The internal structure of dissected animals and cross sections at the positions of the dotted lines are illustrated. Organs other than the mesentery and the digestive tract are ommited. **a** Stage I, just after evisceration. Only the mesentery (gray shaded area) and the cloaca (cl; yellow) remain in the body cavity. **b** Stage II. A regenerating tissue (gut rudiment) appears at the anterior side as a mass of mainly mesenchymal cells surrounded by coelomic epithelium. **c** Stage III. Multiple cavities are formed in the anterior regenerating tissue and these coalesce with each other to form lumens. **d** At stage IV when regeneration is more progressed, the gut rudiment (thickened tissue on the mesentery) becomes continuous between the anterior and posterior sides. The figure shows that the anterior and posterior lumens are not yet connected, but later a single continuous tube is completed. Intestines differentiate according to their position in the digestive tract. In the stomach, a muscle layer (not shown) develops and luminal epithelium (le) is covered with cuticles (cu), as in intact tissues (see Fig. 4a, d). ce: coelomic epithelium; cl: cloaca; cu: cuticles; elt: epithelium-like tissue; le: luminal epithelum

From histological observations, the process of intesitinal regeneration from evisceration (stage I) to completion (stage IV) may be summarized as follows (Fig. 9). At stage I, only the cloaca and the mesentery remain in the body. At stage II, at the anterior end, a thickening which becomes the gut rudiment is formed at the edge of the mesentery; the gut rudiment appears to be composed of mesenchyme with coelomic epithelium surrounding them. At stage III, the intestine starts to regenerate at the posterior end of the body. However, there was a difference in how the lumen is formed between the anterior and posterior gut rudiments. In the anterior region, small multiple cavities/lumens surrounded by epithelium-like tissues arose among the mesenchyme, and the lumen develops by fusing with each other. On the posterior side, a new intestinal tube elongates as a hollow continuous tube from the cloaca, consistent with previous studies in other sea cucumbers (see discussion). At stage IV, the regenerated digestive tract is completed by the coalescence of new tubes generated from the anterior and posterior, and the intestines differentiate.

## Discussion

The present study is the first to report the regeneration of the digestive tract of *Eupentacta quinquesemita* (ishiko) in detail. As reported in other sea cucumber species as well as *E. fraudatrix*, the edge of the mesentery thickens and regenerating tracts expand from both the anterior and posterior rudiments toward the other end of the body. However, our histological observations of the formation of the anterior digestive tube in ishiko differs from what had been reported in the case of *E. fraudatrix*, in which the luminal epithelium is supposed to be formed by invagination of “dedifferentiated mesothelium” [14]. We did not observe any mesothelium penetrating into the mesenchymal cells or invagination. Instead, we found that multiple small cavities are simultaneously formed inside the rudiment, over the period of regeneration, and the lumens fuse with each other, so that the number of lumens decrease and their diameters increase, which eventually results in the formation of a single lumen. The occurrence of multiple epithelia-like cells and cavities in the rudiment at different positions clearly showed that in the anterior gut rudiment, the tube is not formed by simply expanding the lumen or boring a hole inside the rod-like rudiment. In stage IV animals that appeared to have regenerated a continuous gut, the histology revealed that there were parts in the middle of the whole rudiment where tubes were not present or were discontinuous (Fig. 8). Previous investigations in other anterior (*Thyone okeni*) or posterior eviscerating species (*Stichopus mollis*), report that lumina were formed discontinuously and independently [15, 24]. It would be interesting to examine other species whether multiple cavities are formed inside the mesenchyme.

The mechanism by which epithelial tissues arise or cavities/lumens merge to each other remains a question. In the course of the present study, we found that epithelium-like tissues or cells may be distinguished from mesenchyme by color by toluidine blue (TB) staining. TB stains tissues blue, but when the dye binds to acidic sugar chains the color changes to reddish purple or violet. This phenomenon is called metachromasia and is used for detection of acid polysaccharides in cartilage [25]. Staining with TB allowed us to detect multiple epithelium-like tissues differentiating in the anterior gut rudiment, leading to our understanding that multiple cavities are formed within them. We think that this staining method is convenient and effective in studying tube formation in the regenerate, and should be applied to *E. fraudatrix* to compare with ishiko. Furthermore, the metachromasia observed in the anterior gut rudiment suggest that as regeneration/differentiation progresses, differences in the characteristics of extracellular matrix (ECM) emerge. Detection of metachromasia using TB or other stains has also been utilized in previous studies of echinoderms: for example, the presence of acidic or neutral glycosaminoglycans in connective tissue of ishiko was revealed [26], or the mucus of a brittle star, *Ophiocomina nigra*, was determined to be a highly sulfated acid mucopolysaccharide [27].

We note that tube formation in the anterior rudiment resembles secondary neurulation, a process observed in the development of the caudal portion of the spinal cord of amniotes (reviewed in [28, 29]). At the onset of secondary neurulation, mesenchymal cells condense to form a cord-like structure, and lumens are formed inside the compact cord, a process called cavitation. The transformation of mesenchymal cells into an epithelial sheet is known as mesenchymal–epithelial transition (MET) (reviewed in [30, 31]). The formation of the epithelium in secondary neurulation is made possible through MET [32] and MET is also known to be involved in other developmental programs, for example, in somitogenesis [33], kidney development [34], and coelom formation [35]. From our current study, the formation of the epithelial lining of the regenerating lumen of the anterior gut in anterior eviscerating sea cucumbers is added to the example of MET. Mesenchymal–epithelial transition is considered to be a reverse process of epithelial–mesenchymal transition (EMT), and effectors of MET and EMT are supposed to influence each other (reviewed in [30]). As for the molecular mechanism of MET, in some instances such as nephrogenesis, the involvement of signaling molecules are implicated, including cell adhesion molecules (e.g. R-cadherin), growth factors, and transcription factors (reviewed in [31]). It has been reported that N-CAM, a cell adhesion molecule, is one of the candidate molecules implicated in secondary neurulation. This molecule is modified with sialic acid, an acidic sugar, but the intensity of cell adhesion is altered by the quantitative difference in glycosylation [36]. It is our speculation that also during regeneration, changes in the amount and the chemical nature of polysaccharides or sugar chains in the ECM alter the properties of cells to acquire stronger binding to each other to form an epithelium-like tissue, and difference in coloring with TB reflects the differentiation of the cells. It is plausible to think that the molecules involved in MET may be also involved in the process of gut regeneration, and it is a topic that should be looked into to gain a better understanding of the process by which the lumen can be reconstructed from a mass of mesenchymal cells.

## Conclusions

In our study, we were able to elucidate the process of regeneration of the digestive tract in an anterior eviscerating sea cucumber, *Eupentacta quinquesemita* (ishiko). The posterior digestive tube regenerated by forming a continuous lumen from the cloaca. We observed that in the anterior gut rudiment, multiple cavities are formed surrounded by epithelium-like cells, simultaneously and repeatedly, in multiple sites of the rudiment. The cavities inside the rudiment are not formed by invagination from the outer layer of the rudiment; rather, we confirmed that mesenchymal–epithelial transition (MET) is involved. The cavities merge to form lumens, which further coalesce with each other and with the lumen from the posterior, to form a single tube. Staining histological sections with toluidine blue enables distinguishing different cell types and differentiation, and may be a convenient and effective method to further study regeneration of the sea cucumber digestive tract.

## Additional files

Additional file 1: **Figure S1.** Internal morphology of intact *E. quinquesemita*. a View of a dissected and flattened *E. quinquesemita* (left) and a schematic diagram of the organs and tissues (right). The animal was dissected at the right side of the mid-ventral ambulacrum (at the dotted lines in b or c), so the dorsal midline is at the middle of the view and diagram. Five rows of longitudinal muscles (LM) run on the body wall. Gonads, respiratory trees and retractor muscles, etc. that are not relevant to regeneration of the digestive tract are omitted or simplified in the right drawing. b, c Schematic diagrams of cross sections of an animal at the level of the 1st descending intestine (b) and the 2nd descending intestine (c). Arrowheads indicate sites of autotomy at evisceration. Note that the mesentery is attached to the dorsal body wall in b and to the ventral body wall in c. 1 di, 1st descending intestine; ai, ascending intestine; 2di, 2nd descending intestine; es, esophagus; int, intestine; LM: longitudinal muscle, mes, mesentery; OC, oral complex; RT: respiratory tree. (PDF 1963 kb)

Additional file 2: **Figure S2.** Evisceration of *E. quinquesemita.* The oral complex, intestine and gonads are expelled from the hole made by rupturing the anterior end of the body. The digestive tract (dt) exhibits light yellow to orange colors and gonads (go) exhibits yellow-green to green. In this photo, tentacles (T) that are normally folded in the oral complex (OC) is extended and is visible. (PDF 141 kb)

## Data Availability

All data generated or analysed during this study are included in this published article and its supplementary information files.
