# Supplementary figures and images for: Regeneration of the digestive tract of an anterior-eviscerating sea cucumber, Eupentacta quinquesemita, and the involvement of mesenchymal–epithelial transition in digestive tube formation
Source: Zoological Lett. 2019 Jun 21;5:21. doi: 10.1186/s40851-019-0133-3 (PMC6588844; doi:10.1186/s40851-019-0133-3)

Okada and Kondo Supplementary Figure 1

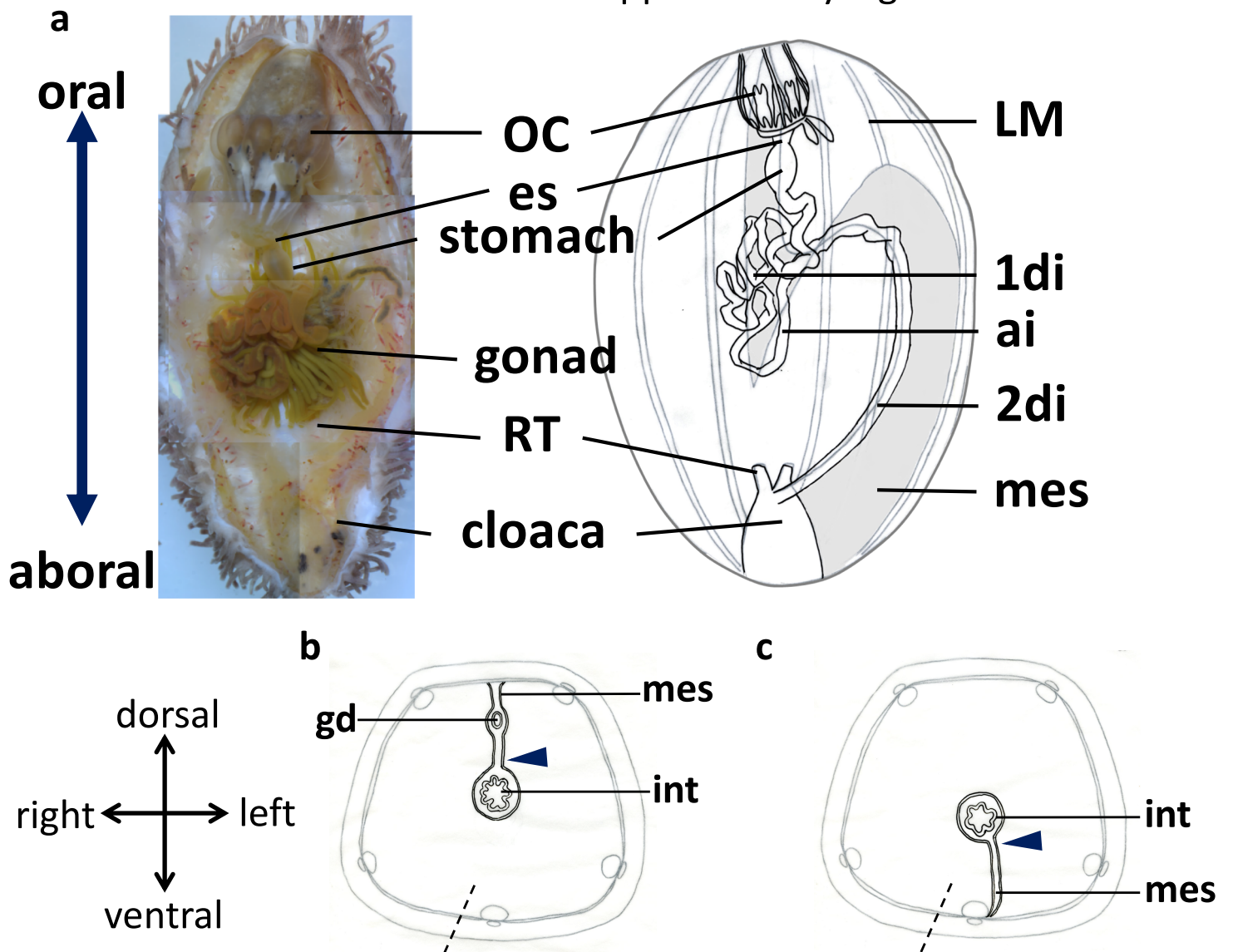

Supplement: Supplementary file 1 — Figure S1. Internal morphology of intact E. quinquesemita. a View of a dissected and flattened E. quinquesemita (left) and a schematic diagram of the organs and tissues (right). The animal was dissected at the right side of the mid-ventral ambulacrum (at the dotted lines in b or c), so the dorsal midline is at the middle of the view and diagram. Five rows of longitudinal muscles (LM) run on the body wall. Gonads, respiratory trees and retractor muscles, etc. that are not relevant to regeneration of the digestive tract are omitted or simplified in the right drawing. b, c Schematic diagrams of cross sections of an animal at the level of the 1st descending intestine (b) and the 2nd descending intestine (c). Arrowheads indicate sites of autotomy at evisceration. Note that the mesentery is attached to the dorsal body wall in b and to the ventral body wall in c. 1 di, 1st descending intestine; ai, ascending intestine; 2di, 2nd descending intestine; es, esophagus; int, intestine; LM: longitudinal muscle, mes, mesentery; OC, oral complex; RT: respiratory tree. (PDF 1963 kb) [file 40851_2019_133_MOESM1_ESM.pdf]

Okada and Kondo Supplementary Figure 2

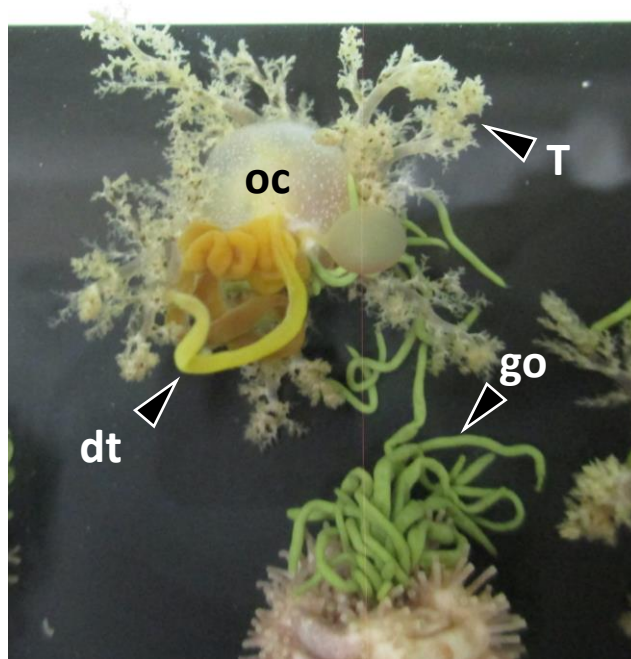

Supplement: Supplementary file 2 — Figure S2. Evisceration of E. quinquesemita. The oral complex, intestine and gonads are expelled from the hole made by rupturing the anterior end of the body. The digestive tract (dt) exhibits light yellow to orange colors and gonads (go) exhibits yellow-green to green. In this photo, tentacles (T) that are normally folded in the oral complex (OC) is extended and is visible. (PDF 141 kb) [file 40851_2019_133_MOESM2_ESM.pdf]
